# Is chronic inflammation a risk factor for perioperative myocardial injury or heart failure in pancreatic surgery patients?

**DOI:** 10.1016/j.bjao.2025.100417

**Published:** 2025-05-19

**Authors:** Ted Reniers, Thijs Rettig, Laura van Zeggeren, Ineke Dijkstra, Kyra Prinsze, Izaak Molenaar, Hjalmar van Santvoort, Olaf Cremer, Lisette Vernooij, Peter Noordzij

**Affiliations:** 1 Department of Anaesthesiology, Intensive Care and Pain Medicine, St Antonius Hospital, Nieuwegein, The Netherlands; 2 Department of Anaesthesiology and Intensive Care, University Medical Centre Utrecht, Utrecht, The Netherlands; 3 Department of Anaesthesiology, Intensive Care and Pain Medicine, Amphia Hospital, Breda, The Netherlands; 4 Department of Anaesthesiology and Pain Medicine, Rijnstate Hospital, Arnhem, The Netherlands; 5 Department of Clinical Chemistry, St Antonius Hospital, Nieuwegein, The Netherlands; 6 Department of Hepato-Pancreato-Biliary Surgery, Regional Academic Cancer Centre Utrecht, St. Antonius Hospital, Nieuwegein, The Netherlands; 7 Department of Hepato-Pancreato-Biliary Surgery, Regional Academic Cancer Centre Utrecht, University Medical Centre Utrecht, Utrecht, The Netherlands

**Keywords:** biomarkers, heart failure, inflammation, pancreatic surgery, PMI

## Abstract

**Background:**

Chronic inflammation is associated with cardiovascular disease. Whether cardiac risk is increased in surgical patients with chronic inflammation is unknown. We hypothesised that preoperative interleukin 6 (IL-6) is associated with postoperative biomarker release indicative of myocardial injury and heart failure.

**Methods:**

In this prospective cohort study in pancreatic surgery patients, concentrations of IL-6, high-sensitive cardiac troponin-T (hs-cTnT), growth differentiation factor 15 (GDF-15), and N-terminal pro B-type natriuretic peptide (NT-proBNP) were assessed before surgery and 4, 12, 24, and 48 h after surgery. The primary outcome was perioperative myocardial injury (PMI), defined as an absolute hs-cTnT increase ≥14 pg ml^−1^. Secondary outcomes were postoperative concentrations of GDF-15 and NT-proBNP. We used the χ^2^ test and generalised linear mixed effects models for analyses.

**Results:**

Of 88 patients, 24 (27%) had high preoperative IL-6 (>7 pg ml^−1^). PMI occurred in two (8.3%) and eight (12.5%) patients with high and normal concentrations, respectively (*P*=0.86). Patients with high IL-6 had higher preoperative concentrations of hs-cTnT (11.0 [inter-quartile range 7.0–15.0] *vs* 8.0 [5.0–11.0] pg ml^−1^, *P*=0.01), GDF-15 (1924.5 [1403.8–2797.5] *vs* 1445.0 pg ml^−1^ [1006.5–1905.3] pg ml^−1^, *P*=0.021) and NT-proBNP (279.5 [128.8–569.0] *vs* 116.5 [65.1–226.5] pg ml^−1^, *P*=0.012). All biomarkers increased after surgery (all *P*<0.05), yet this increase was similar among patients with high or normal preoperative IL-6 concentrations.

**Conclusions:**

Preoperative inflammation was not associated with PMI or postoperative biomarkers of heart failure after pancreatic surgery. However, patients with high IL-6 concentrations had higher preoperative concentrations of cardiac biomarkers, suggesting the presence of subclinical cardiovascular disease.

**Clinical trial registration:**

NCT03460938.

Chronic low-grade inflammation plays an important role in the development of cardiovascular disease and is associated with coronary artery disease and heart failure in the non-surgical population.^1^^,^^2^ Likewise, higher concentrations of preoperative inflammatory biomarkers in surgical patients have been associated with the occurrence of heart disease, specifically perioperative myocardial injury (PMI).^3^, ^4^, ^5^ Biomarkers of inflammation may add value to routine preoperative assessment in identifying (asymptomatic) pre-existing cardiovascular disease. In particular, interleukin 6 (IL-6) has a central role in both initiation and perpetuation of low-grade inflammation, and could be a useful biomarker to improve preoperative risk stratification for PMI and heart failure.

PMI is defined by a perioperative increase of cardiac troponin concentrations and is diagnosed in 15–18% of major noncardiac surgery patients.^6^^,^^7^ Patients with PMI have up to a six-fold increased risk of 30-day postoperative mortality.^8^ About half of these deaths are of cardiovascular origin.^8^ Likewise, patients with acute postoperative heart failure have a poor prognosis. N-terminal pro B-type natriuretic peptide (NT-proBNP) is a biomarker of heart failure that indicates ventricular wall stress and is associated with increased mortality and cardiac events after noncardiac surgery.^9^ Growth differentiation factor 15 (GDF-15) is also associated with heart failure, left ventricular injury, and remodelling and has significant prognostic value in acute heart failure.^10^ This panel of biomarkers thus reflects slightly varying entities of both cardiac injury and heart failure.

The primary aim of or our study was to evaluate the association between preoperative IL-6 and PMI in patients undergoing major noncardiac surgery with high cardiovascular risk. Secondarily, we evaluated the association of preoperative IL-6 with postoperative dynamics of NT-proBNP and GDF-15.

## Methods

### Design and participants

This is a *post hoc* analysis of the myocardial injury and complications after major abdominal surgery (MICOLON 2) study. Full study details have been described previously.^11^ In brief, MICOLON 2 was a single-centre RCT including adult patients undergoing pancreatic surgery (pancreatoduodenectomy, distal pancreatectomy, and total pancreatectomy) between March 2017 and February 2019. Patients were randomly allocated to either remote ischaemic preconditioning (RIPC) or a sham procedure to assess the effect on myocardial injury. No significant difference was observed in the incidence of PMI. The study protocol was approved by the Medical Research Ethics Committees United (MEC-U, number R16.042) and registered before patient enrolment at clinicaltrials.gov (NCT03460938, Principal investigator: P. Noordzij, Date of registration: 09 March 2018). The study was conducted in accordance with the principles of the Declaration of Helsinki. All patients provided written informed consent. This *post hoc* analysis was reported according to the ‘STrengthening the Reporting of OBservational studies in Epidemiology’ (STROBE) checklist.^12^ All patients included in the MICOLON 2 study were considered as having a high surgical-related cardiovascular risk according to the European Society of Cardiology (ESC) guidelines.^6^ The sample size of this *post hoc* analysis was dictated by the sample size of the MICOLON 2 study.

### Biomarkers

Blood samples were collected after induction of general anaesthesia (further referred to as ‘preoperative sample’) and at 4, 12, 24, and 48 h after surgery. The samples were stored at –80°C until analysis. Measurements of hs-cTnT (fifth generation assay), IL-6, GDF-15, and NT-proBNP were performed on an automated Cobas® 8000 platform (Roche Diagnostics, Mannheim, Germany).

Patients were stratified into a normal IL-6 group (preoperative IL-6 concentrations ≤7 pg ml^−1^) and a high IL-6 group (preoperative IL-6 concentrations >7 pg ml^−1^) based on the upper limit of the reference range (95th percentile) for IL-6.^13^ RIPC, the intervention originally studied in this cohort, was not associated with preoperative IL-6 or perioperative hs-cTnT concentrations and therefore not further considered in this analysis.^11^

### Outcomes

The primary outcome was the occurrence of PMI, defined as an absolute increase of ≥14 pg ml^−1^ hs-cTnT (compared with preoperative concentrations) at any time point up to 48 h after surgery, according to the 2022 ESC guidelines.^6^ Secondary analyses were focused on postoperative biomarker dynamics of hs-cTnT, NT-proBNP, and GDF-15 over time. We additionally reported hospital length of stay (days), major adverse cardiovascular events (MACE), and new-onset atrial fibrillation. MACE was defined as a composite of cardiovascular death, myocardial infarction according to the 2022 ESC guidelines, coronary revascularisation, and non-fatal cardiac arrest.^6^

### Statistical analysis

We used χ^2^ test to assess differences in PMI incidence between patients with high and normal preoperative IL-6 concentrations. To investigate the temporal dynamics of hs-cTnT, NT-proBNP, and GDF-15 concentrations, we plotted median values and IQRs of all biomarkers against time. Additionally, generalised linear mixed effects models were constructed to assess the association between preoperative IL-6 concentrations (i.e. coefficient for high *vs* normal IL-6) and perioperative trajectories of biomarkers (i.e. coefficients for time). To assess whether these trajectories differed between patients with high *vs* normal IL-6 concentrations, an interaction term was added. Biomarker values were log transformed to acquire normally distributed residuals. We used a random intercept for each patient and a random slope for time (hours) in all models. As log-GDF-15 trajectory was non-linear, we used a quadratic term for time to improve model fit. We used restricted maximum likelihood estimation to generate unbiased variance estimates. The β-coefficients were calculated and are presented with 95% confidence intervals.

Baseline characteristics are presented as median and inter-quartile range (IQR) for continuous data and numbers with percentages for categorical data. The Mann–Whitney *U* test was used to analyse differences of continuous data and the χ^2^ test for categorical data. A sensitivity analysis of preoperative cardiac biomarker concentrations in patients without cardiovascular disease was performed. A *P*-value of <0.05 was considered statistically significant. All analyses were performed using R version 4.3.1, The R Foundation for Statistical Computing.

## Results

### Participants and preoperative IL-6 concentrations

From the 90 patients who participated in the MICOLON 2 study, two patients (2%) had missing preoperative IL-6 data and were excluded from this analysis. For the remaining patients (*N*=88), there was no missing preoperative biomarker data and at least one postoperative cardiac biomarker measurement was available for each patient. Overall, 10 (2.8%) biomarker values were missing within the 48-h period. The baseline characteristics of the patients are presented in Table 1. The median age was 69 yr (IQR 60.5–72.0) and 37 (42%) patients were female. The Revised Cardiac Risk Index (RCRI) was ≥2 in 30 (34%) patients, and 80 (91%) patients had surgery for cancer. Preoperative IL-6 ranged from 1.5 to 41.7 pg ml^−1^. Furthermore, 24 (27%) patients had preoperative IL-6 concentrations above the upper reference limit of 7 pg ml^−1^, whereas 64 (73%) patients had normal preoperative IL-6 concentrations. No major differences in cardiovascular risk factors were apparent between the groups (Table 1).

**Table 1:** Baseline characteristics of patients with normal and high preoperative IL-6. Continuous values are median (inter-quartile range). Categorical values are numbers (%). Chronic kidney disease is defined as abnormalities of kidney structure or function, present for >3 months, with health implications. Cardiovascular disease is a composite of heart failure, heart valve disease, ischaemic heart disease, peripheral artery disease, and stroke. CRP, C-reactive protein; GDF-15, growth differentiation factor 15; hs-cTnT, high-sensitive cardiac troponin-T; IL-6, interleukin-6; NT-proBNP, N-terminal pro B-type natriuretic peptide; RCRI, Revised Cardiac Risk Index.

Characteristics	Normal IL-6 (≤7 pg ml^−1^)	High IL-6 (>7 pg ml^−1^)	*P-*value

Number of patients	64	24	
Age (yr)	69.5 (56.8–72.0)	69.0 (66.0–73.3)	0.36
Female sex	26 (41)	11 (46)	0.84
BMI	24.8 (22.3–28.2)	24.7 (22.6–26.1)	0.50
Smoking	9 (14)	4 (17)	1.00
Chronic kidney disease	9 (14)	1 (4)	0.36
Cardiovascular disease	19 (30)	7 (29)	1.00
Diabetes mellitus	18 (28)	5 (21)	0.67
Malignancy	57 (89)	23 (96)	0.57
RCRI			0.80
1	41 (64)	17 (71)	
2	15 (23)	5 (21)	
≥3	8 (13)	2 (8)	
Procedure type			0.11
Distal pancreatic resection	11 (17)	1 (4)	
Pancreatoduodenectomy	49 (77)	23 (96)	
Total pancreatectomy	4 (6)	0 (0)	
Biomarkers			
hs-cTnT (pg ml^−1^)	8.0 (5.0–11.0)	11.0 (7.0–15.0)	0.013
NT-proBNP (pg ml^−1^)	116.5 (65.1–226.5)	279.5 (128.8–569.0)	0.012
GDF-15 (pg ml^−1^)	1445.0 (1006.5–1905.3)	1924.5 (1403.8–2797.5)	0.021
CRP (mg l^−1^)	3.7 (2.0–6.2)	29.6 (22.2–48.1)	<0.001

### Preoperative IL-6 and perioperative myocardial injury

Ten patients (11%) met the criteria for PMI (i.e. an absolute hs-cTnT increase ≥14 pg ml^−1^ compared with preoperative concentrations), 2 (8%) in the high IL-6 group and 8 (13%) in the normal IL-6 group, which was not statistically different (difference between proportions; 4% [95% CI –13 to 21, *P*=0.86]). Patients with PMI were older, more often men, and more frequently diagnosed with cardiovascular and chronic kidney disease (Supplementary Table S1).

**Fig 1:**
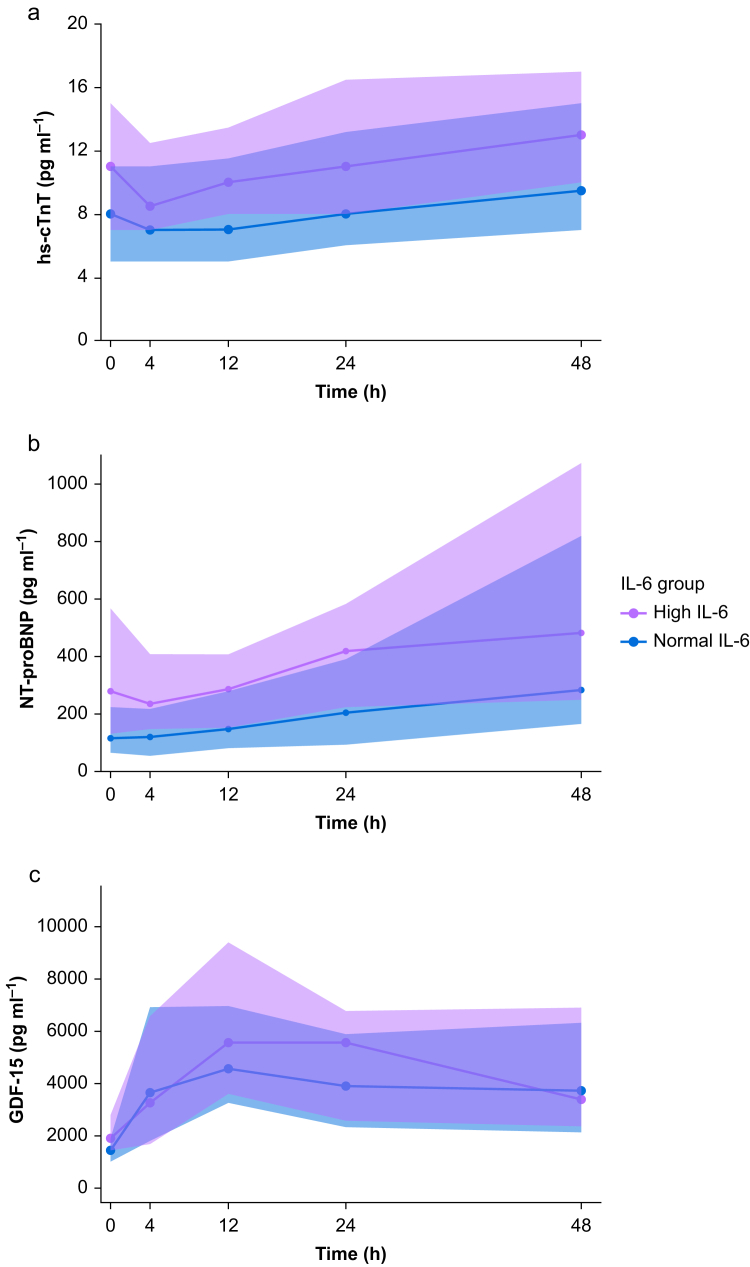
Perioperative biomarker concentrations. Median (inter-quartile range) of (a) high-sensitive cardiac troponin-T (hs-cTnT), (b) N-terminal pro B-type natriuretic peptide (NT-proBNP), (c) and growth differentiation factor 15 (GDF-15) concentrations at all perioperative time points stratified by preoperative interleukin-6 (IL-6) group.

Patients with high preoperative IL-6 concentrations had higher preoperative hs-cTnT concentrations (11.0 pg ml^−1^ [IQR 7.0–15.0] *vs* 8.0 pg ml^−1^ [5.0–11.0], *P*=0.01). Postoperative hs-cTnT peaked 48 h after surgery in 69% of patients and median hs-cTnT concentrations were 13.0 pg ml^−1^ (10.0–17.0) and 9.5 pg ml^−1^ (7.0–15.0) in the high and normal IL-6 groups, respectively (*P*=0.07; Fig. 1). Overall, log hs-cTnT concentrations increased in the postoperative period (β for time [hours]: 0.008, 95% CI [0.00–0.01], *P*=<0.001). However, this increase was similar in both groups (β for interaction [IL-6 group and time]: –0.001, 95% CI [–0.009 to 0.006], *P*=0.75; Supplementary Table S2).

### Preoperative IL-6 and biomarkers of heart failure

Figure 1 shows the trajectory of NT-proBNP and GDF-15 over the first 48 h after surgery according to normal or high preoperative IL-6. Preoperative NT-proBNP concentrations were higher in the high IL-6 group (279.5 [128.8–569.0] *vs* 116.5 pg ml^−1^ [65.1–226.5], *P*=0.012; Table 1). After surgery, peak concentrations of NT-proBNP occurred after 48 h (482.0 pg ml^−1^ [248.5–1070.5] in the high IL-6 group *vs* 283.5 pg ml^−1^ [165.3–819.5] in the normal IL-6 group, *P*=0.12). Log NT-proBNP concentrations increased after surgery (β 0.023, *P*<0.001). The postoperative increase of log NT-proBNP concentrations did not differ between groups, (β –0.004, *P*=0.43; Supplementary Table S2).

Preoperative GDF-15 concentrations were higher in the high IL-6 group (1924.5 pg ml^−1^ [1403.8–2797.5] *vs* 1445.0 pg ml^−1^ [1006.5–1905.3], *P*=0.021; Table 1). Peak concentrations of GDF-15 were reached at 12 h after surgery (5566.0 pg ml^−1^ [3597.5–9412.5] in the high IL-6 group *vs* 4555.0 pg ml^−1^ [3239.0–6956.0] in the normal IL-6 group, *P*=0.32). The perioperative trajectory of log GDF-15 concentrations was not affected by the preoperative IL-6 concentrations (β –0.001, *P*=0.8; Supplementary Table S2).

### Preoperative subclinical cardiovascular disease

In a subgroup of patients without diagnosed cardiovascular disease, preoperative cardiac biomarker concentrations were higher in patients with high preoperative IL-6 concentrations (Supplementary Table S3).

### Preoperative IL-6 and clinical outcomes

The median length of hospital stay was 16 days (IQR 10–21) and 13 days (8–20) in patients with high- and normal preoperative IL-6, respectively (*P*=0.30). One patient in the normal IL-6 group had a MACE, and three patients developed new-onset atrial fibrillation. In the high IL-6 group, MACE and new-onset atrial fibrillation did not occur (*P*=1.00 and *P*=0.68, respectively).

## Discussion

In patients undergoing pancreatic surgery, preoperative inflammation was not a risk factor for PMI, nor was it associated with biomarkers of heart failure or cardiac remodelling.

Our results shed new light on the association between preoperative inflammation and PMI.^4^ A previous study demonstrated that an elevated preoperative neutrophil lymphocyte ratio (NLR) was associated with PMI. NLR is a biomarker that combines information about the innate immune response, characterised by neutrophils, and the adaptive immune response, characterised by lymphocytes. The authors suggested that elevated preoperative NLR, reflecting systemic inflammation, may contribute to PMI. In contrast to our study, PMI was defined as hs-cTnT concentration of ≥14 pg ml^−1^ within 3 days after surgery, without taking preoperative hs-cTnT values into account. Our results suggest that increased postoperative hs-cTnT concentrations in patients with chronic inflammation are a consequence of higher preoperative hs-cTnT concentrations, instead of PMI.

The increased concentrations of preoperative hs-cTnT, NT-proBNP, and GDF-15 in patients with high preoperative IL-6 suggest the presence of subclinical cardiovascular disease. In a systematic review and meta-analysis, the incremental value of preoperative troponin and NT-proBNP concentrations to the RCRI for the prediction of MACE and mortality was assessed.^14^ The RCRI is a cardiac risk model in noncardiac surgery comprising clinically apparent cardiovascular risk factors. It provides the preoperative probability of in-hospital MACE. Both troponin and NT-proBNP improved prediction of MACE and mortality when added to the RCRI, suggesting the clinical relevance of subclinical cardiovascular disease indicated by these biomarkers. Although preoperative inflammation may identify patients with subclinical cardiovascular disease, it remains unknown whether inflammatory biomarkers have incremental value compared with cardiac biomarkers alone regarding cardiovascular risk assessment.

Patients with high preoperative IL-6 concentrations had postoperative NT-proBNP concentrations exceeding the cut-off of 300 pg ml^−1^ used for the diagnosis of acute heart failure according to the ESC.^15^ To diagnose acute heart failure, a combination of clinical symptoms and elevated NT-proBNP concentrations is needed, which was not assessed in our study. Still, regardless of clinical symptoms, isolated increased postoperative NT-proBNP concentrations are associated with increased risk for MACE and mortality and these patients could theoretically benefit from treatment.^9^ The incremental value of preoperative inflammatory biomarkers for cardiac risk management needs to be further explored.

### Strengths and limitations

An important strength of this study is the use of the latest PMI definition from the ESC guideline 2022 with both preoperative and postoperative hs-cTnT measurements up to 48 h after surgery.^6^ Prospective biomarker collection resulted in only 10 (2.8%) missing biomarker values over the 48-h postoperative period. Additionally, we provide new insight into the perioperative trajectories of two biomarkers of heart failure and cardiac remodelling (NT-proBNP and GDF-15) in patients with or without preoperative inflammation.

Our study has several limitations. A type II error may have occurred as we analysed a small cohort of pancreatic surgery patients with a relatively low incidence of PMI. We cannot rule out that significant differences would have been shown with a larger sample size. In our cohort, 11% of patients met the criteria for PMI. In 69% of patients, hs-cTnT concentrations were still increasing 48 h after surgery. Extending the blood collection period to ≥3 days might have resulted in a higher PMI incidence. Ideally, we would have included potential confounders such as age and comorbidities in the analysis, but this was prevented by the small sample size. Finally, IL-6 is a biomarker with a rapid response and rather short half-life, raising the question whether it is a suitable marker to identify a chronic inflammatory process.^16^ If there is, however, a chronic and active inflammatory process, IL-6 concentrations would conceivably be elevated.

In conclusion, preoperative IL-6 was not associated with PMI or biomarkers associated with heart failure in patients who underwent pancreatic surgery. There were, however, increased concentrations of preoperative hs-cTnT, NT-proBNP, and GDF-15 in patients with high preoperative IL-6. This suggests the presence of subclinical cardiovascular disease in patients with preoperative chronic inflammation. Further research on the combined use of preoperative inflammatory and cardiac biomarkers to evaluate cardiac risk is needed to improve perioperative risk stratification.

## Authors’ contributions

Conceptualisation: T Reniers, T Rettig, OC, PN

Methodology: LV

Data acquisition: ID

Investigation: LZ

Data interpretation: KP, IM, HS, OC, PN

Project administration: LZ

Formal analysis: T Reniers

Supervision: PN

Writing – original draft: T Reniers, PN

Writing – review and editing: LZ, ID, KP, IM, HS, OC, LV

Critical revision: T Rettig

Final approval: all authors

## Funding

Funding for biomarker analysis was provided by Roche Diagnostics. Roche Diagnostics had no role in design and conduct of the study, analysis and interpretation of the data, or preparation and approval of the manuscript.

## Declaration of interests

PN is a member of the advisory board of Roche Diagnostics on the perioperative use of biomarkers. PN, T Rettig, and T Reniers conduct a separate research study on perioperative biomarkers funded by 10.13039/100016545Roche Diagnostics. All other authors have no conflicts of interest.
