# Supplementary material for: Is chronic inflammation a risk factor for perioperative myocardial injury or heart failure in pancreatic surgery patients?
Source: BJA Open. 2025 May 19;14:100417. doi: 10.1016/j.bjao.2025.100417 (PMC12145987; doi:10.1016/j.bjao.2025.100417)
Supplement: Multimedia component 1 [file mmc1.docx]

**Supplementary Material**

Supplementary Table S1. Baseline characteristics stratified by PMI

| **Characteristics** | **No PMI** | **PMI** | **p-value** |
| --- | --- | --- | --- |
| Number of patients | 78 | 10 |  |
| Age, yr | 69.00 (58.25, 72.00) | 70.50 (68.25, 74.00) | 0.241 |
| Female sex | 36 (46) | 1 (10) | 0.066 |
| BMI | 24.80 (22.62, 27.87) | 22.90 (22.20, 26.60) | 0.419 |
| Smoking | 10 (13) | 3 (30) | 0.333 |
| Chronic Kidney Disease | 7 ( 9) | 3 (30) | 0.149 |
| Cardiovascular disease | 21 (27) | 5 (50) | 0.255 |
| Diabetes Mellitus | 20 (26) | 3 (30) | 1.000 |
| Malignancy | 70 (90) | 10 (100) | 0.633 |
| RCRI |  |  | 0.843 |
| 1 | 52 (67) | 6 (60) |  |
| 2 | 17 (22) | 3 (30) |  |
| ≥3 | 9 (12) | 1 (10) |  |
| Procedure type |  |  | <0.001 |
| Distal pancreatic resection | 9 (12) | 3 (30) |  |
| Pancreatoduodenectomy | 68 (87) | 4 (40) |  |
| Total pancreatectomy | 1 (1) | 3 (30) |  |

Supplementary Table S2. Estimates Mixed Effects Model analyses

| **Log hs-cTnT ~ time + high IL-6 + time*high IL-6** |  |  |  |
| --- | --- | --- | --- |
| Variable | β | 95% CI | p-value |
| Intercept | 2.1 | (2.0 - 2.3) | <0.001 |
| time | 0.01 | (0.00 - 0.01) | <0.001 |
| high IL-6 | 0.30 | (-0.03 - 0.64) | 0.08 |
| time:high IL-6 | -0.00 | (-0.01 - 0.01) | 0.75 |
| **Log NT-proBNP ~ time + high IL-6 + time*high IL-6** |  |  |  |
| Variable | β | 95% CI | p value |
| Intercept | 4.8 | (4.5 – 5.0) | <0.001 |
| time | 0.02 | (0.02 – 0.03) | <0.001 |
| high IL-6 | 0.61 | (0.06 – 1.2) | 0.03 |
| time:high IL-6 | -0.00 | (-0.01 – 0.01) | 0.43 |
| **Log GDF-15 ~ time + time^2 + high IL-6 + time*high IL-6** |  |  |  |
| Variable | β | 95% CI | p-value |
| Intercept | 7.7 | (7.5 - 7.8) | <0.001 |
| time | 0.06 | (0.05 - 0.07) | <0.001 |
| time^2 | -0.00 | (-0.00 -0.00) | <0.001 |
| high IL-6 | 0.17 | (-0.17 - 0.51) | 0.31 |
| time:high IL-6 | -0.00 | (-0.01 - 0.01) | 0.76 |

Supplementary Table S3. Preoperative biomarker concentrations in patients without cardiovascular disease

| **Characteristics** | **Normal IL-6 (≤7 pg/ml)** | **High IL-6 (>7 pg/ml)** | **p value** |
| --- | --- | --- | --- |
| Number of patients | 45 | 17 |  |
| hs-cTnT (pg/ml) | 8.00 (5.00, 9.00) | 10.00 (7.00, 12.00) | 0.03 |
| NT-proBNP (pg/ml) | 99.80 (48.60, 137.00) | 184 (107, 559) | 0.02 |
| GDF-15 (pg/ml) | 1226 (976, 1799) | 1525.00 (1393, 2157) | 0.09 |
| hs-cTnT ≥ 14 pg/ml N (%) | 5 (11.1) | 3 (17.6) | 0.80 |
| NT-proBNP ≥300 pg/ml N (%) | 5 (11.1) | 7 (41.2) | 0.02 |

**Supplementary Legends**

Supplementary Table S1. Continuous values are median (interquartile range). Categorical values are numbers (%). BMI, Body Mass Index, RCRI, Revised Cardiac Risk Index, PMI, Perioperative Myocardial Injury. Chronic kidney disease is defined as abnormalities of kidney structure or function, present for >3 months, with health implications. Cardiovascular disease is a composite of heart failure, heart valve disease, ischemic heart disease, peripheral artery disease and stroke.

Supplementary Table S2. Estimates of Linear mixed effects models. Time in hours. CI, confidence interval, hs-cTnT, high sensitive cardiac troponin-T, NT-proBNP, N-terminal pro B-type natriuretic peptide, GDF-15, growth differentiation factor 15, IL-6, Interleukin-6.

Supplementary Table S3. Continuous values are median (interquartile range). Categorical values are numbers (%). Abbreviation: Hs-cTnT, high sensitive cardiac troponin-T, NT-proBNP, N-terminal pro B-type natriuretic peptide, GDF-15, growth differentiation factor 15, IL-6, Interleukin-6.
